# Are mirrors aversive or rewarding for mice? Insights from the mirror preference test

**DOI:** 10.3389/fnbeh.2023.1137206

**Published:** 2023-04-13

**Authors:** Shigeru Watanabe

**Affiliations:** Department of Psychology, Keio University, Minato-Ku, Tokyo, Japan

**Keywords:** mirror, preference, anxiety, stress, mirror chamber test

## 1. Introduction

Visual stimulation *via* mirror images has been examined among various animals, including fish, birds, rodents, monkeys, and great apes. Most of these species demonstrate social or aggressive behavior toward mirrors. Although mice have poor vision and predominantly use olfaction to gather information from the surrounding environment, visual stimulation through mirrors appears to be effective also in this species. Research investigating the effects of mirror exposure in mice found that the presence of mirrors has similar effects to the presence of cage mates. Restraint in a small holder induces hyperthermia (stress-induce hyperthermia: SIH) in mice but a restrained mouse surrounded by similarly restrained cage mates shows less SIH (Watanabe, 2015). A restrained mouse surrounded by mirrors instead of the cage mates also shows reduced SIH, suggesting that the images reflected by the mirrors are a substitute for conspecifics (Watanabe, 2016). However, there is mixed evidence on the effect of mirrors on mice. In a study on chronic mirror-image stimulation, Fuss et al. (2013) found that a mirror placed for 5 weeks in the cage of single-housed mice had no effect on anxiety and depression-like behaviors. Nevertheless, the presence of the mirror increased exploratory behavior, enhancing both rearing in the novel cage exploration test and head-dipping in the hole-board test. Conversely, pharmacological studies have used mirrors to induce anxiety in mice.

This suggests that mirrors have contrasting effects on mice. In the present article, we will examine mirror-based rodent behavioral tests and compare their individual characteristics to understand the effect of mirrors on mice. Moreover, we will describe under which conditions mirrors could be used as rewards. Indeed, mirror-based behavioral tests would be particularly useful for behavioral neuroscience research. Since the mirror reward does not require previous starvation or water deprivation, it would be an animal-friendly alternative to classical appetitively motivated learning tests employing food or water as reward. Refinement of current behavioral tests is important both to maximize animal welfare and to reduce stress-associated variability, hence improving reproducibility of scientific results (d'Isa and Gerlai, 2023).

## 2. Mirror chamber test to examine anxiety in mice

### 2.1. Design of a mirror chamber test

**Figure 1:**
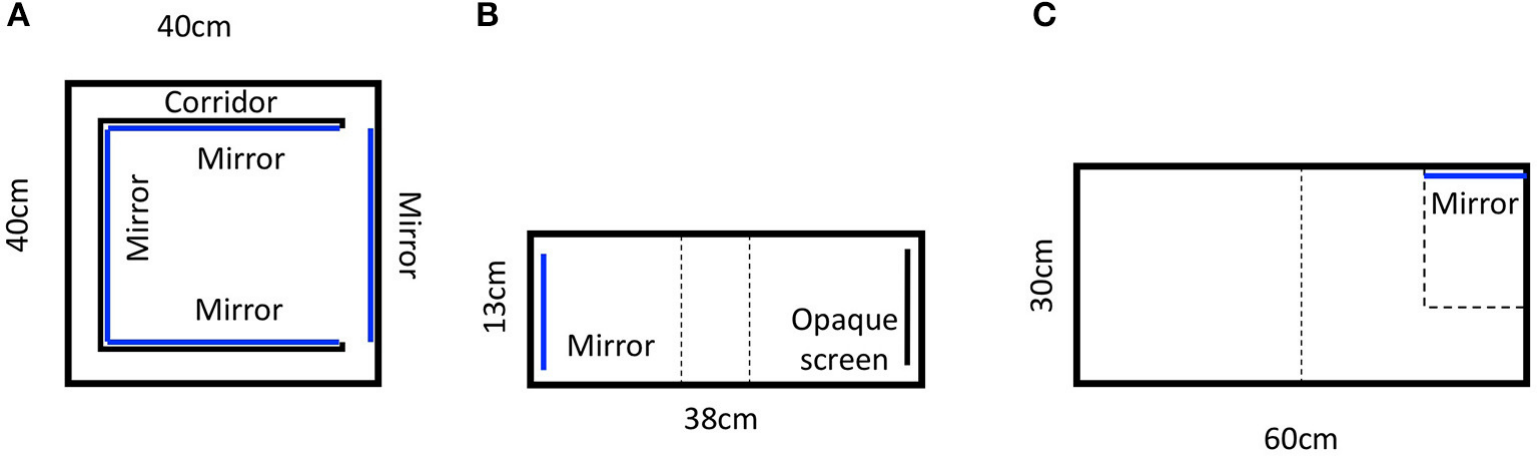
Apparatus of mirror experiments. **(A)** Toubas et al. (1990), **(B)** Watanabe (2016), **(C)** Ueno et al. (2020). Blue lines represent mirrors.

Various methods have been employed to measure anxiety in rodents, including the elevated plus maze, the light-dark box test, the conflict test and the social defeat test (see Belzung and Griebel, 2001; Parle et al., 2010). As a method alternative to the elevated plus-maze test, Toubas et al. (1990) invented the mirror chamber test, which consisted of a mirrored cube, open on one side, that was placed in a square Plexiglas box (Figure 1A). This cube (30 cm × 30 cm × 30 cm) was constructed from five pieces of mirrored glass (three side panes, a top pane, and a floor pane) with a single open side. The mirror chamber was placed in a container box (40 cm × 40 cm × 30.5 cm). A sixth mirror was placed on the container wall and positioned such that it faced the single open side of the mirrored chamber. The container thus formed a 5 cm corridor surrounding the mirrored chamber. Mice (Balb/c) were placed in the corner of the corridor and allowed to move around the container for 30 min. Latency to enter the mirrored chamber was used as an index of anxiety. The authors injected the mice with different doses of diazepam, a known anxiolytic, and found that the latency decreased in a dose-dependent manner. This evidence indicates that the mirror chamber test is an effective tool in pharmacological studies. The authors claimed that the method was simple, non-punishing, rapid, and quantitative.

### 2.2. Behavioral indexes of anxiety

Alongside latency measures of anxiety, other methods included measuring the number of entries into the mirrored chamber and the total time spent in the mirrored chamber (Reddy and Kulkarni, 1997; Paterson et al., 2010). Toubas et al. (1990) measured the number of animals that exhibited a latency longer than 200s. Kliethermes et al. (2003) employed a modified mirror chamber without a ceiling, placed mice inside the chamber as start position, and used voluntary re-entry time (defined as the total time spent in the mirror chamber minus the initial latency to exit the chamber). These studies reported agreement between conventional latency measurements and alternative indices.

### 2.3. Control conditions

Various control conditions have been employed in mirror studies. For example, Toubas et al. (1990) used an inverted mirror chamber, where the corridors had mirrors (instead of the chamber) and found a significant difference in mean latencies between entering the mirror chamber (1039s) and the inverted mirror chamber (14s). The short latency in entering the inverted mirror chamber may reflect an avoidance of mirrored corridors. Similar findings were obtained by Lamberty (1998), who compared the latency between three chambers: a mirror chamber, a white chamber, and a gray chamber. Balb/c and DBA mice avoided these three chambers, while C57BL/6 mice spent a longer time in the gray chamber than in the white and mirror chambers. The author suggested that brightness may explain the avoidance of mirror and white chambers. In this case, the avoidance of mirrors would not derive from aversion toward the images of the mirror, but rather from aversion toward the strong light reflected by the mirror, related to mice's natural photophobia.

## 3. Mirror preference test

In a two-choice test of mirror preference (Sherwin, 2004), C57BL/6 mice were placed in an apparatus comprising two cages connected by a tunnel. They occupied the mirror cage 47.6% of the time demonstrating no significant differences between preferences for mirror and non-mirror conditions. Similarly, in a study of mirror preference, Fuss et al. (2013) observed for 5 min how much time C57BL/6 mice spent in the chambers. Consistent with Sherwin's (2004) findings, mice did not demonstrate any preference or aversion to the mirrors. More recent studies innovated the mirror preference test by using two successive tests rather than simultaneous choices of preference. For example, Ueno et al. (2020) used an apparatus that was divided into two areas (Figure 1B). After overnight exposure to a mirror in their home cages, the researchers measured time spent by C57BL/6 mice in the central empty area and in board areas for 20 min. Subsequently, the mirror was placed in the board area and the time spent in each area was measured again, thus examining mirror preference using two successive tests. The mice spent approximately the same amount of time in areas with and without the mirror, demonstrating a consistent lack of preference for, or avoidance of, mirrors.

Using a similar two-choice place-preference experiment design, Yakura et al. (2018) tested rats' preferences for mirrors. They utilized an elaborate apparatus with an empty chamber on one end and a mirror, a video-recorded image of a rat, or a still image of a rat at the opposite end (Yakura et al., 2018). The rats spent significantly more time in the mirror chamber and video-recorded image chamber than in their respective empty chambers. Analogously, Watanabe (2016) used a two-choice place preference apparatus (Figure 1C) to test how much time C57BL/6 mice spent in the mirror compartment vs. the compartment with an opaque screen, when they were observed for 10 min. Notably, 17 of the 24 mice demonstrated significant preferences for the mirror, contradicting the findings of Sherwin (2004) and Ueno et al. (2020).

There are several procedural differences between these studies and Watanabe's (2016) study. Firstly, Sherwin (2004) repeatedly measured preferences every 10 min for 24 h, while Ueno et al. (2020) compared mirror and non-mirror preferences by using successive tests. Secondly, the illumination used by Watanabe (2016) was relatively low (11.0 lux). Unfortunately, the luminance of the apparatus in other experiments is not known. However, mice are naturally photophobic (i.e., they are averse to bright light) and this aversion may have masked a possible mirror preference. Another confound in the studies by Sherwin (2004) and Ueno et al. (2020) is that mirrors tend to appear much brighter than control objects in well-lit environments due to their reflective nature. This could have elicited an aversion to the mirrors, masking the mice's preference for them.

## 4. Mirrors vs. live animals

Watanabe (2016) also demonstrated that mice preferred mirrors over unfamiliar live mice, but not over familiar live mice (cage mates). Their aversion to unfamiliar mice, and similar preferences for mirrors and cage mates contrasts with the findings of Ueno et al. (2020). In this simultaneous presentation test with a mirror and a stranger (enclosed in a transparent cage), mice showed a preference for the stranger, demonstrating that they were able to discriminate between unfamiliar conspecifics and mirrors. It is important to note that in this experiment, the stranger mice were placed in transparent cages endowed with holes that were 1 cm in diameter. This allowed visual, tactile and olfactory stimulation. Nose contact and sniffing may have driven the preference for chambers with unfamiliar conspecifics. Since olfaction is a primary sense in mice, olfactory curiosity may be a stronger motivator than visual curiosity. This behavior has been observed in another study that employed a perforated partition to separate an unfamiliar conspecific from subject mice. The mice displayed approach behavior and spent longer time in its proximity (Harda et al., 2022). Conversely, Watanabe (2016) separated the subject mice *via* a transparent partition without holes, which excluded the possibility of proximal investigation through nose contact and sniffing of the unfamiliar conspecific. By removing the possibility of physical contact and confounds arising from olfaction, and focusing on visual stimulation, the method of Watanabe (2016) allowed to evaluate more accurately the ability of the subject mice to discriminate between the visual appearance of an unfamiliar conspecific and the visual image reflected in the mirror. Thus, this paradigm provides a less biased comparison between the effects of mirrors and unfamiliar conspecific exposure.

## 5. What is measured in the mirror chamber test?

In the mirror chamber test, mice avoided the mirror chamber, suggesting that the presence of the mirror had an anxiogenic effect. However, it is important to carefully assess and distinguish the causes of this anxiety. For instance, there is evidence that mirror placement could be crucial. Watanabe (2016) placed mirrors only on the side walls of the chamber, reflecting natural and realistic images of conspecifics, and this had an anti-stress effect on mice. In contrast, in the mirror chamber test mirrors were placed on the side wall, the floor and the ceiling. Mirrors placed in these positions reflect unusual and unnatural images of conspecifics, which may induce anxiety in mice. Another critical confounding factor in the mirror chamber test is the narrow corridor around the mirror chamber. Mice display a tendency known as thigmotaxis, or wall-hugging, which is a preference for narrow spaces. They have an aversion to open spaces and prefer to stay close to lateral barriers. The behavior of mice in the mirror chamber test is the summation of two factors: first, the effect of being exposed for the first time to unnatural images of conspecifics; second, aversion for open spaces and consequent thigmotaxis. Moreover, as the measurement of mirror preference (or aversion) is sensitive to procedural details, such as behavioral adaptation to the mirror, time of testing, and lighting conditions, these methodological details should be standardized for pharmacological testing. In addition to the procedural differences, difference in strain of experimental subjects also affects the results.

Importantly, under unbiased conditions, where these confounds are controlled by employing equally sized and shaped chambers, natural reflected images and low luminosity, mirrors appear to be rewarding for mice and have a positive effect on their affective state.

## 6. Mirrors as rewards

The reward value of a stimulus (i.e., its effectiveness as a conditioning stimulus) can be evaluated through a two-choice preference test. Research demonstrated that bright lights and unnatural reflected images are anxiogenic factors that can produce bias in the preference test and hence should be avoided. However, under unbiased conditions (natural reflected images and low luminosity), mirrors appear to be rewarding for mice, that show a clear preference for mirrors over opaque control objects (Watanabe, 2016). Additionally, mirrors showed an anti-stress effect on mice, as revealed by non-invasive infrared thermography assessment of stress-induced hyperthermia (Watanabe, 2016). Considering these results, it is possible that mirrors might be effective reinforcers in conditioning paradigms. Conditioning can be of two types. Respondent (classical or Pavlovian) conditioning features the establishment of an association between two stimuli (conditioned and unconditioned stimuli), whereas operant (instrumental or Skinnerian) conditioning features a contingency of three events, stimulus (discriminative stimulus), behavior (operant) and a reinforcer (d'Isa et al., 2011). Respondent conditioning can be assessed through the conditioned place preference (CPP) test (Tzschentke, 2007) and the conditioned place aversion (CPA) test (Schechter and Meechan, 1994). CPP and CPA have been used mostly in pharmacological studies but aversive state without drug injection has also been employed, for example, water-flood induced CPA in mice (Goltseker and Barak, 2018). To test the potential of mirrors as a reward in respondent conditioning, mice could be trained in a CPP apparatus with two chambers: on one side, a chamber with a vertically-striped wall facing a wall with attached a flat opaque object; on the other side, a chamber with a horizontally-striped wall facing a wall with attached a flat mirrored object (equal in size to the opaque object). The experimental subject is allowed to stay in one chamber on day one and in the other chamber on day two. After repeating this conditioning procedure, the subject will undergo a test. In the test session, the two objects should be removed, along with the partition separating the two chambers, and mice will be allowed to move freely across the chambers. Preference between the vertically striped and horizontally striped chambers will provide an index of the rewarding value of the mirror. Indeed, non-pharmacological place conditioning procedures are particularly useful, since the drugs commonly used in the ordinal pharmacological CPP can interfere with memory and learning (Goltseker and Barak, 2018).

On the other hand, the reinforcing value of a mirror in operant conditioning can be assessed by measuring, for example, lever pressing to access a mirror. The strength of its reinforcing value should be measured by a progressive ratio schedule in which the number of responses required to receive reinforcement is gradually increased until the subject stops responding.

Mirror-based tests could become a new class of animal-friendly learning tests. Indeed, mirrors could be employed as animal-friendly reinforcers to study learning and memory processes. Investigation of mirror-induced conditioning could lead to the development of new behavioral tests that do not require punishment or prior stressful conditions, such as food or water deprivation.

## Author contributions

SW conceived and wrote the manuscript, created the figures, and acquired funding.

## References

[B1] Belzung C. Griebel G. (2001). Measuring normal and pathological anxiety-like behavior in mice: a review. Behav. Brain Res. 125, 141–149. 10.1016/S0166-4328(01)00291-1 11682105

[B2] d'Isa R. Gerlai R. (2023). Designing animal-friendly behavioral tests for neuroscience research: the importance of an ethological approach. Front. Behav. Neurosci. 16, 1090248. 10.3389/fnbeh.2022.1090248 36703720 PMC9871504

[B3] d'Isa R. Solari N. Brambilla R. (2011). “Biological memory in animals and in man,” in Memory Mass Storage, eds Campardo G. Tiziani F. Iaculo M. (Berlin-Heidelberg: Springer), 417–441. 10.1007/978-3-642-14752-4_9

[B4] Fuss J. Richter S. H. Steinle J. Deubert G. Hellweg R. Gass P. (2013). Are you real? Visual simulation of social housing using mirror image stimulation in single-housed mice. Behav. Brain Res. 243, 191–198. 10.1016/j.bbr.2013.01.015 23333841

[B5] Goltseker K. Barak S. (2018). Flood-conditioned place aversion as a novel non-pharmacologicalaversive learning procedure in mice. Sci. Rep. 8, 7280. 10.1038/s41598-018-25568-5 29740070 PMC5940895

[B6] Harda Z. Misiołek K. Klimczak M. Chrószcz M. Rodriguez Parkitna J. (2022). C57BL/6N mice show sub-strain-specific resistance to the psychotomimetic effects of ketamine. Front. Behav. Neurosci. 16, 1057319. 10.3389/fnbeh.2022.1057319 36505728 PMC9731130

[B7] Kliethermes C. L. Finn D. A. Crabbe J. C. (2003). Validation of the modified mirrored chamber sensitive to anxiolytics and anxiogenics in mice. Psychopharmacology 69, 190–197. 10.1007/s00213-003-1493-z 12783153

[B8] Lamberty Y. (1998). Mirror chamber test for anxiolytics: Is there a mirror-induced stimulation? Physiol. Behav. 64, 703–705. 10.1016/S0031-9384(98)00124-3 9817584

[B9] Parle M. Devi S. Kumar S. (2010). Animal models for screening anxiolytic agents. Ann. Pharm. Pharm. Sci. 1, 116–128.

[B10] Paterson N. E. Iwunze M. Davis S. F. Malekiani S. A. Taleen Hanania T. (2010). Comparison of the predictive validity of the mirror chamber and elevated plus maze tests in mice. J. Neurosci. Meth. 188, 62–70. 10.1016/j.jneumeth.2010.02.005 20149823

[B11] Reddy D. S. Kulkarni S. K. (1997). Differential anxiolytic effects of neurosteroids in the mirrored chamber behavior test in mice. Brain Res. 752, 61–71. 10.1016/S0006-8993(96)01447-3 9106441

[B12] Schechter M. D. Meechan S. M. (1994). Conditioned place aversion produced by dopamine release inhibition. Eur. J. Pharmacol. 260, 133–137. 10.1016/0014-2999(94)90329-8 7988636

[B13] Sherwin C. M. (2004). Mirrors as potential environmental enrichment for individually housed laboratory mice. Appl. Anim. Behav. Sci. 87, 95–103. 10.1016/j.applanim.2003.12.01426221832

[B14] Toubas P. L. Abla K. A. Cao W. Logan L. G. Seale T. W. (1990). Latency to enter a mirrored chamber: a novel behavioral assay for anxiolytic agents. Pharmacol. Biochem. Behav. 35, 121–126. 10.1016/0091-3057(90)90215-4 2315349

[B15] Tzschentke T. M. (2007). Measuring reward with the conditioned place preference (CPP) paradigm: update over the last decade. Addict. Biol. 12, 227–462. 10.1111/j.1369-1600.2007.00070.x 17678505

[B16] Ueno H. Suemitsu S. Murakami S. Kitamura N. Wani K. Takahashi Y.. (2020). Behavioral changes in mice after becoming accustomed to the mirror. Behav. Neurol. 2020, 4071315. 10.1155/2020/4071315 32089750 PMC7023847

[B17] Watanabe S. (2015). Social inequality enhances stress-induced hyperthermia in ICR mice. Brain Res. 1624, 134–139. 10.1016/j.brainres.2015.07.019 26232073

[B18] Watanabe S. (2016). Mirror perception in mice: preference for and stress reduction by mirrors. Internal. J. Comp. Psychol. 29, 10. 10.46867/ijcp.2016.29.00.10

[B19] Yakura T. Yokota H. Ohmichi Y. Ohmichi M. Nakano T. Naito M. (2018). Visual recognition of mirror, video-recorded, and still images of rats. PLoS ONE 13, e0194215. 10.1371/journal.pone.0194215 29534087 PMC5849344

